# Minimal reaction schemes for pattern formation

**DOI:** 10.1098/rsif.2023.0490

**Published:** 2024-02-28

**Authors:** Fraser R. Waters, Christian A. Yates, Jonathan H. P. Dawes

**Affiliations:** ^1^ Department of Mathematical Sciences, University of Bath, Bath BA2 7AY, UK; ^2^ Centre for Mathematical Biology, University of Bath, Bath BA2 7AY, UK

**Keywords:** pattern formation, reaction scheme, Turing pattern, mass-action kinetics

## Abstract

We link continuum models of reaction–diffusion systems that exhibit diffusion-driven instability to constraints on the particle-scale interactions underpinning this instability. While innumerable biological, chemical and physical patterns have been studied through the lens of Alan Turing's reaction–diffusion pattern-forming mechanism, the connections between models of pattern formation and the nature of the particle interactions generating them have been relatively understudied in comparison with the substantial efforts that have been focused on understanding proposed continuum systems. To derive the necessary reactant combinations for the most parsimonious reaction schemes, we analyse the emergent continuum models in terms of possible generating elementary reaction schemes. This analysis results in the complete list of such schemes containing the fewest reactions; these are the simplest possible hypothetical mass-action models for a pattern-forming system of two interacting species.

## 1. Introduction

The spontaneous emergence of structure is a widely recognized and central aspect of spatially distributed complex systems, relevant to fields from cosmology [1] to polymer physics [2,3] to biology [4,5]. Turing's strikingly simple model for spontaneous pattern formation in a system of two chemical species via the interplay between reaction and diffusion continues to have a defining influence on the field [6–8]. Natural patterns that have been analysed using Turing's instability include digit formation in vertebrates [9,10], fingerprint formation [11], animal skin pigmentation patterning [12,13], non-equilibrium chemical dynamics [14,15] and vegetation distributions [16]. However, very few of the popular and widely used canonical mathematical pattern-forming models, such as those associated with Prigogine & Lefever [17], Schnakenberg [18] and Gray & Scott [19], correspond directly (without invoking additional assumptions) to a set of chemical reactions involving individual particles. Such model equations therefore cannot immediately be used to generate direct illustrations of particle-scale chemical reaction schemes that correspond to continuum models undergoing a Turing instability. To remedy this situation, here we derive, for the first time, the complete list of the most parsimonious reaction schemes which do this (i.e. that lead to continuum models which are able to exhibit Turing patterns).

**Figure 1.**
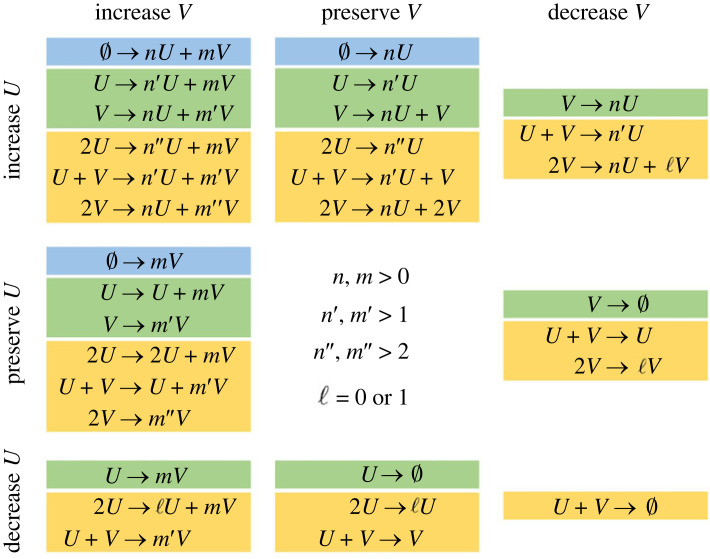
Classification of elementary reactions for two species into 31 qualitatively different kinds. Rows and columns separate reactions into those that increase, preserve, or decrease the number of particles of species *U* and *V* respectively. Interactions that do not change either *U* or *V* cannot be considered to be meaningful reactions; hence in the central grid element we list the various constraints on the integer numbers of particles produced (i.e. the stoichiometric coefficients) which are designated *n*, *n*′, *n*″, *m*, *m*′, *m*″ and ℓ. Reactions have been coloured according to the number of reacting particles (the reaction ‘order') blue, zeroth order; green, first order; yellow, second order.

Although there are 31 qualitatively different individual reactions up to second order (figure 1), and therefore several thousand possible combinations of sets of three or four of them, we show that out of this plethora of possibilities there are in fact only 25 such qualitatively distinct minimal reaction schemes. Intriguingly, we find that two-species Turing patterns in which the concentration peaks are spatially in-phase can result from sets of only three reactions, while, in contrast, four reactions are required to generate patterns in which the concentration peaks are in spatial anti-phase. This reveals a new fundamental difference between these two cases, which hitherto have been presented as equally complex alternatives. Our new catalogue establishes, in terms of chemical reactions, the precise conditions under which Turing patterns form, links particle-scale and continuum models directly, explains the minimal ingredients required and informs the design of sensible stochastic simulations for pattern formation. As a whole, it provides a new level of fundamental clarity and insight into pattern formation that applies across scientific domains.

From a historical perspective, our motivation in grounding Turing instabilities in elementary reactions follows in Turing's own footsteps [6,20,21]. By ‘elementary', we mean that we consider only zeroth-, first- or second-order (pseudo-)reactions [22], where we equate the order of the reaction with the number of reacting particles. For example, the zeroth-order reaction ∅→nU should be interpreted as the supply of *n* particles of reagent *U* from a large reservoir where the supply rate is constant. Our focus on elementary reactions excludes mass-action laws that contain cubic or higher-order nonlinearities, or non-polynomial terms: cubic terms would naturally be interpreted as trimolecular interactions, while non-polynomial terms in reaction equations can arise from attempting to model systems containing ad hoc reaction rates, catalysts, saturation effects or the formation of complexes (e.g. through dimerization or trimerization) that equilibrate on much faster time scales; these can often be derived under limiting assumptions from larger systems with polynomial reaction terms of the kind we consider, but they are not in themselves minimal examples by our definition. Also following Turing, we consider diffusion to be isotropic, reaction kinetics to be well described by mass-action laws (for example, omitting finite-size effects [23]), and reactions to involve only two chemical species. Given these natural simplifying assumptions, we achieve a complete enumeration of the minimal models for Turing patterns. This catalogue reveals the fundamental necessary and sufficient ingredients for Turing instability, at the particle scale, and thereby provides new insights into pattern formation via reaction–diffusion mechanisms.

In §2, we introduce the general form of partial differential equation (PDE) reaction–diffusion model which will be our analytical starting point along with its relation to underlying particle interactions, and we present the classical conditions for such a PDE model to exhibit Turing instability. In §3, we leverage the Turing instability conditions to derive simple stoichiometric constraints on the types of reactions present in the underlying reaction scheme, such that the corresponding PDE model can exhibit Turing instability for some choice of rate- and diffusivity-parameter values. In §4, we complete the analysis for reaction schemes of few reactions exhibiting one particular type of Turing instability: we derive sufficient stoichiometric constraints on the reaction scheme such that the corresponding PDEs exhibit Turing instability for appropriate choices of parameter values, and we present a classification of all such schemes up to a qualitative equivalence which is described in §2. We also discuss the reduction, in terms of the linearized non-dimensionalized dynamics, of all of these classes to a single regime diagram, which is shown in figure 5.

## 2. Model set-up and definitions

We consider two populations of particles, labelled *U* and *V*, which react according to reactions ${\left\{R_{i}\right\}}_{i=1}^{N}$ each described symbolically in the standard way,

2.1 $$R_{i}:\;p_{i}U+q_{i}V\overset{r_{i}}{⟶}n_{i}U+m_{i}V,$$

where *r_i_* > 0 is the reaction rate parameter, *p_i_*, *q_i_* are the (non-negative) integer stoichiometric reactant coefficients, and *n_i_*, *m_i_* are the (non-negative) integer stoichiometric product coefficients. We define the net stoichiometric effects $s_{i1}:=n_{i}-p_{i}$, $s_{i2}:=m_{i}-q_{i}$, and note that *s_i_*_1_ ≥ −*p_i_* and *s_i_*_2_ ≥ −*q_i_*. Such a set of reactions ${\left\{R_{i}\right\}}_{i=1}^{N}$ constitutes a *reaction scheme*, and the combined effects of the individual reactions and diffusion yields a PDE model for the space- and time-dependent concentrations *u*(*x*, *t*) and *v*(*x*, *t*). Supposing that reactions take place locally in space, between large numbers of particles of *U* and *V* in a manner such that mass-action laws apply, and that diffusion is isotropic, the generic form that such PDEs take is

2.2a $$\partial_{t}u=D_{u}\nabla^{2}u+F(u,\;v)$$

and

2.2b $$\partial_{t}v=D_{v}\nabla^{2}v+G(u,\;v),$$

where

$$F(u,\;v)=\underset{i}{\sum }r_{i}s_{i1}u^{\;p_{i}}v^{q_{i}},\;\;\;\;\;G(u,\;v)=\underset{i}{\sum }r_{i}s_{i2}u^{\;p_{i}}v^{q_{i}}.$$

Confining attention to elementary (i.e. at most bimolecular) reactions, we restrict *p_i_* + *q_i_* ≤ 2, and thus the reaction terms take the form of quadratic polynomials

2.3a $$F(u,\;v)=a_{1}+a_{2}u+a_{3}v+a_{4}u^{2}+a_{5}uv+a_{6}v^{2}$$

and

2.3b $$G(u,\;v)=b_{1}+b_{2}u+b_{3}v+b_{4}u^{2}+b_{5}uv+b_{6}v^{2},$$

which are subject to a number of constraints, both implicit and explicit. The major implicit constraint is that for the model to be well-posed, non-negative initial conditions must always yield non-negative concentrations at later times. Explicitly, of the 12 coefficients {*a_j_*, *b_j_*} six must be non-negative: *a*_1_, *a*_3_, *a*_6_ > 0 and *b*_1_, *b*_2_, *b*_4_ > 0 due to the constraints (respectively) *s_i_*_1_ ≥ −*p_i_* and *s_i_*_2_ ≥ −*q_i_* for each reaction *R_i_*.

To the authors' knowledge, there appear to be only two published models of Turing instability that use a mass-action reaction scheme having only quadratic nonlinearities [24,25]. The first of these (by Levin and Segel [24]) is essentially an extension of the well-known Lotka–Volterra system and corresponds to the coefficient choices *a*_2_, *a*_4_, *b*_5_ > 0 and *a*_5_, *b*_6_ < 0, with all other coefficients set to zero. The second (by Woolley, Krause and Gaffney [25]) is given as one of two sets of PDEs for which a pattern emerges in a specific subset of parameter space.

The Turing instability conditions, as we shall consider them, for the PDE system (2.2)–(2.3) are well known and we summarize them as follows:

1. The system supports a positive spatially uniform steady state: there exist positive concentrations u* > 0, v* > 0 satisfying *F*(u*, v*) = *G*(u*, v*) = 0.
2. The uniform steady state is linearly stable to spatially uniform perturbations: under the assumption of spatial uniformity, the Jacobian evaluated at the steady state,
  $$J^{*}=\left(\begin{array}{cc} J_{11}^{*} & J_{12}^{*}\\ J_{21}^{*} & J_{22}^{*} \end{array}\right):={\left(\begin{array}{cc} \partial_{u}F & \partial_{v}F\\ \partial_{u}G & \partial_{v}G \end{array}\right)|}_{\left(u,v)=(u^{*},v^{*}\right)},$$
must satisfy the Routh–Hurwitz linear stability criteria tr(J*) < 0 and det(J*) > 0
  2.4 $$J_{11}^{*}+J_{22}^{*}<0\;$$
and
  2.5 $$J_{11}^{*}J_{22}^{*}-J_{12}^{*}J_{21}^{*}>0.$$
3. The uniform steady state is linearly unstable to spatially non-uniform perturbations. Including diffusion and expanding a spatial perturbation $\left(\tilde{u},\;\tilde{v}\right)$ in the eigenbasis of the diffusion operator, in one spatial dimension this requires that the wavenumber-dependent Jacobian,
  $$\tilde{J}(k):=J^{*}-k^{2}\left(\begin{array}{cc} D_{u} & 0\\ 0 & D_{v} \end{array}\right),$$
must satisfy $\det(\tilde{J}(k))<0$ for some *k* > 0, (since $\mathrm{tr}(\tilde{J}(k))\leq$ $\mathrm{tr}(J^{*})<0$). Written out explicitly, this inequality takes the form
  2.6 $$\begin{array}{rl} & D_{u}D_{v}k^{4}-(J_{11}^{*}D_{v}+J_{22}^{*}D_{u})k^{2}+(J_{11}^{*}J_{22}^{*}-J_{12}^{*}J_{21}^{*})<0\;\\ & \;\;\;\;\;\mathrm{for}\;\mathrm{some}\;k>0. \end{array}$$
We note that in higher spatial dimensions, *k*^2^ is replaced by |***k***|^2^ where ***k*** is the wavevector of the plane wave perturbation to the spatially uniform state.

A necessary condition for inequality (2.6) to be satisfied is that $J_{11}^{*}D_{v}+J_{22}^{*}D_{u}>0$, from which—in conjunction with the trace condition (2.4)—we deduce that exactly one of $J_{11}^{*}$ and $J_{22}^{*}$ must be positive and the other negative. Thus, we recast condition (2.5) as the stricter necessary condition

2.7 $$J_{12}^{*}J_{21}^{*}<J_{11}^{*}J_{22}^{*}<0,$$

from which we infer that $J_{12}^{*}$ and $J_{21}^{*}$ have different signs. In particular, inequality (2.7) implies the established result [5] for two-component Turing systems that the signs of the entries of J* must match one of the following patterns:

$$J^{*}∼\left(\begin{array}{cc} + & -\\ + & - \end{array}\right),\left(\begin{array}{cc} + & +\\ - & - \end{array}\right),\left(\begin{array}{cc} - & +\\ - & + \end{array}\right)\;\mathrm{or}\;\left(\begin{array}{cc} - & -\\ + & + \end{array}\right).$$

We draw attention to the commonality between these four cases that, at the uniform steady-state concentrations (u*, v*), to leading order one species behaves as an auto-activator and the other as an auto-inhibitor, i.e. the diagonal elements have opposite signs. That is, either

$${\frac{\partial }{\partial u}F(u,v)|}_{\left(u^{*},v^{*}\right)}>0\;\;\;\mathrm{and}\;\;\;{\frac{\partial }{\partial v}G(u,v)|}_{\left(u^{*},v^{*}\right)}<0,$$

or

$${\frac{\partial }{\partial u}F(u,v)|}_{\left(u^{*},v^{*}\right)}<0\;\;\;\mathrm{and}\;\;\;{\frac{\partial }{\partial v}G(u,v)|}_{\left(u^{*},v^{*}\right)}>0.$$

By swapping the labels *U* and *V*, without loss of generality we may consider just the first of these two cases.

We refer to a reaction scheme (2.1) as *Turing-unstable* if the corresponding PDE model (2.2)–(2.3) admits a positive steady state (u*, v*) at which the Jacobian J* satisfies conditions (2.4) and (2.7) for some reaction rate parameters {*r_i_*} in an open subset of $R_{+}^{N}$. These together guarantee that condition (2.6) holds for diffusivities (*D_u_*, *D_v_*) in an open subset of $R_{+}^{2}$ given by

2.8 $$\sqrt{\frac{D_{v}}{D_{u}}}>\sqrt{\delta_{c}}=\frac{1}{J_{11}^{*}}\left(\sqrt{\det(J^{*})}+\sqrt{-J_{12}^{*}J_{21}^{*}}\;\right).$$

The unstable wavenumbers are those in the interval *k*_−_ < *k* < *k*_+_, where

2.9 $$k_{\pm }^{2}=\frac{D_{v}J_{11}^{*}+D_{u}J_{22}^{*}\pm \sqrt{{\left(D_{v}J_{11}^{*}+D_{u}J_{22}^{*}\right)}^{2}-4D_{u}D_{v}\det(J^{*})}}{2D_{u}D_{v}},$$

and the corresponding perturbations have linearized growth rate *λ* given by

2.10 $$\begin{array}{r} \lambda (k^{2})=\frac{1}{2}(J_{11}^{*}+J_{22}^{*}-(D_{u}+D_{v})k^{2} \end{array}\;+\sqrt{{\left((D_{v}-D_{u})k^{2}+J_{11}^{*}-J_{22}^{*}\right)}^{2}+4J_{12}^{*}J_{21}^{*}}\;).$$

In an infinite spatial domain, the above Turing instability conditions are sufficient for the homogeneous steady state (u*, v*) to be rendered unstable to spatial perturbations: for short times the state loses stability to a dominant sinusoidal waveform with wavenumber *k* that maximizes the real part of the growth rate *λ*. In practice, on finite domains boundary criteria impose selection mechanisms on the set of possible wavenumbers, which can prevent the instability if no possible wavenumber lies in the interval (*k*_−_, *k*_+_). However, for simplicity we do not consider this effect in this paper except implicitly in relation to our numerical simulations.

We also note that instability of the homogeneous steady state does not at all imply asymptotic stability of a regular spatio-temporal patterned state at nearby parameter values. Further, various other dynamical effects and secondary bifurcations may also be manifest at later times; while important in gaining a fuller understanding of the implications of our results these also are not explored in this paper and are likely to vary in subtle ways across the sets of parameter values within each minimal scheme, and from one minimal scheme (with its specific PDE model) to another.

We will classify reaction schemes in the following way. We say that two reactions *R_i_*, *R_j_* are *qualitatively equivalent* if (*p_i_*, *q_i_*, sign(*s_i_*_1_), sign(*s_i_*_2_)) = (*p_j_*, *q_j_*, sign(*s_j_*_1_), sign(*s_j_*_2_)). Otherwise, we consider two reactions to be *qualitatively distinct*. This yields 31 qualitatively distinct types of reactions that are of second-order or lower; these are summarized in the stoichiometric equations displayed in figure 1. We say that two reaction schemes $S_{1}={\left\{R_{i}^{\left(1\right)}\right\}}_{i=1}^{N}$, $S_{2}={\left\{R_{i}^{\left(2\right)}\right\}}_{i=1}^{N}$ are *qualitatively equivalent* if, up to some reordering of the reactions, $R_{i}^{\left(1\right)}$ is qualitatively equivalent to $R_{i}^{\left(2\right)}$ for each *i* = 1, … , *N*. In this paper, we seek to enumerate the classes of reaction schemes (up to the above qualitative equivalence) that admit any Turing-unstable reaction schemes, under the restriction that the *size* of the reaction scheme (i.e. the number of reactions *N*) be as small as possible. Our analysis presents a systematic and complete exploration of when the PDEs (2.2) satisfy the Turing instability conditions (2.4) and (2.7) under suitable constraints on the coefficients of the reaction kinetics, with the ‘minimality' of schemes imposing a constraint on the number of non-zero coefficient pairs (*a_i_*, *b_i_*).

## 3. Necessary but insufficient reactions

**Figure 2.**
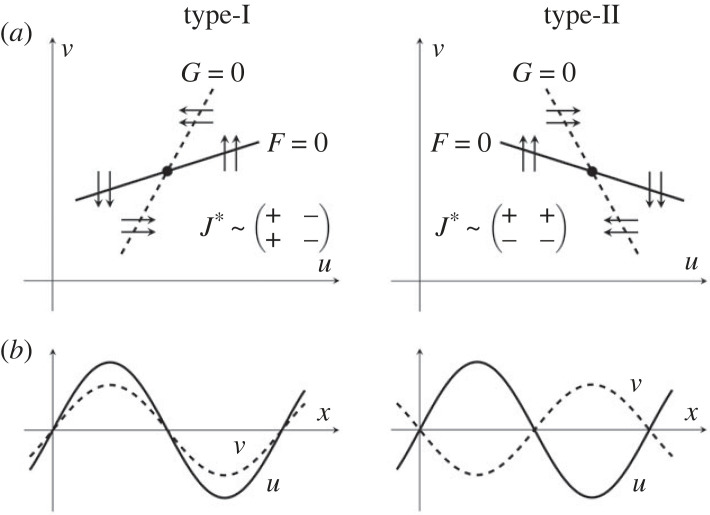
Two types of Turing patterns: spatially in-phase or anti-phase. (*a*) Phase plane schematics for the non-spatial dynamics of systems close to a steady state that supports Turing patterns of type I (in-phase, left) or type II (anti-phase, right). Solid and dashed lines, respectively, mark the nullclines ∂*_t_u* = 0 and ∂*_t_v* = 0; arrows signify the flow of solutions (*u*(*t*), *v*(*t*)). (*b*) Illustrative spatial profiles of *u*(*x*, *t*) (solid lines) and *v*(*x*, *t*) (dashed lines) for type-I and type-II patterns.

It is well known [25,26] that there are two distinct types of Turing patterns for two species: type I (in which concentration peaks are spatially aligned—‘in-phase'), also referred to as ‘pure activator–inhibitor' dynamics, and type II (in which peaks in one species correspond to troughs in the other—‘anti-phase’), also referred to as ‘cross activator–inhibitor' dynamics, figure 2*b*. The form of interactions between the species when the system is close to the spatially homogeneous steady state determines which pattern type arises in any particular model. Mathematically, the entries in the Jacobian matrix J* (i.e. the linearization of the reaction polynomials *F*(*u*, *v*) and *G*(*u*, *v*) around the uniform equilibrium) must have one of the following two sign patterns:

3.1a $$\mathrm{type}\;I:\;J^{*}∼\left(\begin{array}{cc} + & -\\ + & - \end{array}\right)$$

and

3.1b $$\mathrm{type}\;\mathrm{II}:\;J^{*}∼\left(\begin{array}{cc} + & +\\ - & - \end{array}\right).$$

The linearized dynamics in the phase space, ignoring the spatial diffusion terms, are then mirror images of each other, shown schematically in figure 2*a*.

Despite the apparent symmetry between the linearized dynamics, type-I and type-II patterns are in fact fundamentally different in terms of required particle interactions. Type-II patterns are more complex, and this is revealed by elucidation of the particle reactions necessary to generate them. Supposing the existence of a positive uniform steady state (u*, v*), the general form for the non-spatial Jacobian is

$$J^{*}=\left(\;\begin{array}{cc} J_{11}^{*} & J_{12}^{*}\\ J_{21}^{*} & J_{22}^{*} \end{array}\right)=\left(\begin{array}{cc} a_{2}+2a_{4}u^{*}+a_{5}v^{*} & a_{3}+a_{5}u^{*}+2a_{6}v^{*}\\ b_{2}+2b_{4}u^{*}+b_{5}v^{*} & b_{3}+b_{5}u^{*}+2b_{6}v^{*} \end{array}\right).$$

Combining the conditions for Turing instability gives constraints on the signs of some of the coefficients {*a_i_*, *b_i_*}, necessitating that reactions of certain qualitative types be included in the reaction scheme. For type-I patterns there are two necessary reactant combinations, for type-II patterns there are three, which we now describe.

### 3.1. Reactions involving two *U* particles

For both type-I and type-II patterns, a necessary condition is $J_{11}^{*}>0$. Using the steady-state equation for *u*,

$$0=F(u^{*},\;v^{*})=a_{1}+a_{2}u^{*}+a_{3}v^{*}+a_{4}{\left(u^{*}\right)}^{2}+a_{5}u^{*}v^{*}+a_{6}{\left(v^{*}\right)}^{2},$$

we may rewrite

$$J_{11}^{*}=a_{2}+2a_{4}u^{*}+a_{5}v^{*}=a_{4}u^{*}-\frac{1}{u^{*}}(a_{1}+a_{3}v^{*}+a_{6}{\left(v^{*}\right)}^{2}).$$

Since *a*_1_, *a*_3_ and *a*_6_ are non-negative, positivity of $J_{11}^{*}$ requires *a*_4_ > 0. This requires that at least one reaction with reactant combination 2*U* and a positive stoichiometric effect on species *U* be included in the reaction scheme.

### 3.2. Reactions involving one particle of each species

For type-I patterns, a necessary condition is $J_{12}^{*}=a_{3}+a_{5}u^{*}+2a_{6}v^{*}<0$. Since *a*_3_ and *a*_6_ are non-negative, negativity of $J_{12}^{*}$ requires *a*_5_ < 0. This requires that at least one reaction with reactant combination *U* + *V* and a negative stoichiometric effect on species *U* be included in the reaction scheme. For type-II patterns, a necessary condition is $J_{21}^{*}=b_{2}+2b_{4}u^{*}+b_{5}v^{*}<0$. Since *b*_2_ and *b*_4_ are non-negative, negativity of $J_{21}^{*}$ requires *b*_5_ < 0. This requires that at least one reaction with reactant combination *U* + *V* and a negative stoichiometric effect on species *V* be included in the reaction scheme.

In each case, a reaction with reactant combination *U* + *V* must be included in the reaction scheme. Further, if only one of *a*_5_ and *b*_5_ is negative—i.e. if the net contribution of all reactions with reactant combination *U* + *V* decreases the number of particles of only one species—then we can deduce the type of the pattern: if *a*_5_ < 0 ≤ *b*_5_ then it is of type I, if *a*_5_ ≥ 0 > *b*_5_ then it is of type II.

### 3.3. Reactions involving one *U* particle

For type-II patterns, $J_{11}^{*}>0$ and $J_{12}^{*}>0$ are both necessary conditions. We may compute that

$$\begin{array}{rl} u^{*}J_{11}^{*}+v^{*}J_{12}^{*} & =a_{2}u^{*}+a_{3}v^{*}+2a_{4}{\left(u^{*}\right)}^{2}+2a_{5}u^{*}v^{*}+2a_{6}{\left(v^{*}\right)}^{2}\\ & =2F(u^{*},v^{*})-(2a_{1}+a_{2}u^{*}+a_{3}v^{*})\\ & =-2a_{1}-(a_{2}u^{*}+a_{3}v^{*}). \end{array}$$

Since *a*_1_ and *a*_3_ are non-negative, simultaneous positivity of $J_{11}^{*}$ and $J_{12}^{*}$ requires *a*_2_ < 0. This requires that at least one first-order reaction with reactant *U* and a negative stoichiometric effect on species *U* be included in the reaction scheme.

Thus, a reaction scheme for type-I patterns requires at least two specific reactant combinations: the reaction scheme must contain reactions of the form 2*U* → ⋯ and *U* + *V* → ⋯, whereas a reaction scheme for type-II patterns requires at least three: 2*U* → ⋯, *U* + *V* → ⋯ and *U* → ⋯. We now show that, in order to satisfy all the necessary conditions for Turing instability, at least one further reaction is required in both cases.

*Type I:* For a scheme of reactions with only reactant combinations 2*U* and *U* + *V*, the interaction terms are

$$F(u,\;v)=a_{4}u^{2}+a_{5}uv,$$

and

$$G(u,\;v)=b_{4}u^{2}+b_{5}uv.$$

Assuming the existence of a positive steady state (u*, v*), using the steady-state equations we may rewrite the Jacobian

$$J^{*}=\left(\begin{array}{cc} 2a_{4}u^{*}+a_{5}v^{*} & a_{5}u^{*}\\ 2b_{4}u^{*}+b_{5}v^{*} & b_{5}u^{*} \end{array}\right)=\left(\begin{array}{cc} -a_{5}v^{*} & a_{5}u^{*}\\ -b_{5}v^{*} & b_{5}u^{*} \end{array}\right),$$

yielding det(J*) = 0. This violates the necessary condition that (u*, v*) be strictly linearly stable to spatially uniform perturbations.

*Type II:* For a scheme of reactions with only reactant combinations 2*U*, *U* + *V* and *U*, the interaction terms are

$$F(u,\;v)=a_{2}u+a_{4}u^{2}+a_{5}uv,$$

and

$$G(u,\;v)=b_{2}u+b_{4}u^{2}+b_{5}uv.$$

Assuming the existence of a positive steady state (u*, v*), using the steady-state equations we may rewrite the Jacobian

$$J^{*}=\left(\;\begin{array}{cc} a_{2}+2a_{4}u^{*}+a_{5}v^{*} & a_{5}u^{*}\\ b_{2}+2b_{4}u^{*}+b_{5}v^{*} & b_{5}u^{*} \end{array}\;\right)=\left(\;\begin{array}{cc} a_{4}u^{*} & a_{5}u^{*}\\ b_{4}u^{*} & b_{5}u^{*} \end{array}\;\right),$$

and thus $J_{21}^{*}\geq 0$ (since *b*_4_ is necessarily non-negative). This violates the necessary condition for type-II patterns that $J_{21}^{*}<0$.

Hence, overall a type-I pattern requires at least three distinct reactions, while a type-II pattern requires at least four reactions. What we refer to as *minimal schemes* for each pattern type are those Turing-unstable reaction schemes comprising the smallest number of reactions. We will demonstrate that for type-I patterns it is sufficient to have three reactions, while for type-II patterns it is sufficient to have four reactions, and thus these are, respectively, the sizes of the minimal schemes for patterns of types I and II.

## 4. Minimal schemes

**Figure 3.**
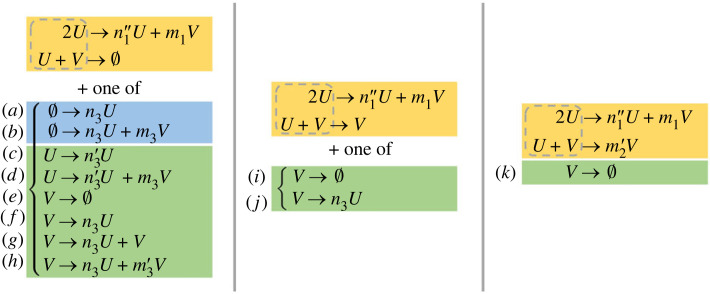
The 11 qualitatively distinct classes of reaction schemes that contain the fewest number (three) of elementary reactions and admit Turing patterns of type I. Each class is contained within a separate column and left braces indicate a choice of reactions, so that the left-hand column comprises eight distinct classes, the central column two classes, and the right-hand column a single class. Following the convention set out in figure 1, the stoichiometric coefficients are labelled with apostrophes to indicate different constraints: *n_j_*, *m_j_* must be positive, $n_{j}^{\prime },\;m_{j}^{\prime }>1$, and $n_{j}^{\prime \prime },m_{j}^{\prime \prime }>2,$ for any *j*. Hence in every case the 2*U* → · · · reaction must increase the numbers of particles of both *U* and *V*, while the *U* + *V* → · · · reaction must decrease the number of particles of *U*. Moreover, the third reaction cannot decrease the number of particles of *U*. Reactions have been coloured according to the number of reacting particles (the reaction ‘order') blue, zeroth order; green, first order; yellow, second order.

**Figure 4.**
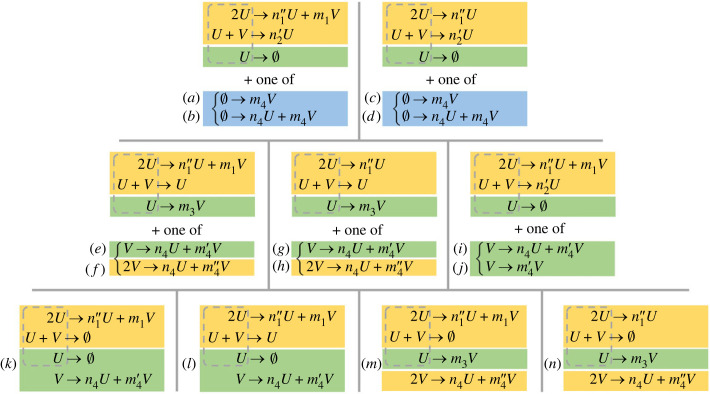
The 14 qualitatively distinct classes of reaction schemes of four elementary reactions admitting Turing patterns of type II. Classes are contained within a grid box and left braces indicate a choice of one out of a pair of possible reactions, leading to two distinct classes in each of the five boxes in the upper rows. Following the convention set out in figure 1, the stoichiometric coefficients are labelled with apostrophes to indicate different constraints: *n_j_*, *m_j_* must be positive, $n_{j}^{\prime },\;m_{j}^{\prime }>1$ and $n_{j}^{\prime \prime },m_{j}^{\prime \prime }>2,$ for any *j*. Hence in every case the 2*U* → · · · reaction must increase the number of particles of *U*, while the *U* + *V* → · · · reaction must decrease the number of particles of *V*. The third reaction must decrease the number of particles of *U*. The fourth reaction must increase the number of particles of *V* and cannot decrease the number of particles of *U*. Reactions have been coloured according to the number of reacting particles (the reaction ‘order') blue, zeroth order; green, first order; yellow, second order.

An exhaustive analysis of reaction schemes that satisfy the conditions for Turing instability using the fewest number of reactions yields 11 qualitatively distinct minimal schemes for patterns of type I (which we derive in detail in this section) and 14 qualitatively distinct minimal schemes for patterns of type II (derived in electronic supplementary material, text section A). These minimal schemes are summarized in figures 3 and 4, respectively. In a separate analysis, for each scheme, we compute the parameter values for the diffusion coefficients *D_u_* and *D_v_* for which a Turing instability arises. In every case, we find an open set of parameter values which demonstrates that the instability is structurally robust in the sense that it will generically persist in the presence of small perturbations to the reaction rates and diffusion coefficients.

There are four options for reactants for a third reaction (with a different reactant combination) in addition to the necessary 2*U* → · · · and *U* + *V* → · · · reactions for type-I patterns: ∅, *U*, *V* or 2*V*. We work through each of these possibilities in turn, either deriving the constraints on the qualitative reaction types or checking that the reaction scheme is not Turing-unstable. It transpires that, for all minimal schemes for type-I patterns, the Jacobian determinant and sign conditions (2.6), (3.1) amount to constraints on the stoichiometric product coefficients only, while the Jacobian trace condition (2.3) imposes a constraint on the reaction rate parameters. In total, we find 11 classes of qualitatively distinct reaction schemes that contain *some* schemes satisfying the conditions for a Turing pattern instability. These are presented in figure 3, and their derivation comprises the bulk of this section.

For type-II minimal schemes, the analysis becomes more complicated since there are often two positive homogeneous steady states, only one of which can undergo a Turing instability, and there is a third dimensionless parameter group, formed from the reaction rate constants. Further details and commentary are provided in the electronic supplementary material, text section D.

### 4.1. Third reaction of order zero

If we choose the third reaction to be of zeroth order, existence of a positive steady state requires that the bimolecular *U* + *V* reaction must also decrease the number of particles of species *V*, determining the qualitative type of this reaction. We may then write our reaction scheme as

$$\{\begin{array}{c} \;2U\overset{r_{1}}{\to }n_{1}U+m_{1}V\\ \;U+V\overset{r_{2}}{\to }\emptyset \\ \emptyset \overset{r_{3}}{\to }n_{3}U+m_{3}V \end{array},$$

with corresponding reaction terms

$$F(u,\;v)=r_{3}n_{3}+r_{1}(n_{1}-2)u^{2}-r_{2}uv\;$$

and

$$G(u,\;v)=r_{3}m_{3}+r_{1}m_{1}u^{2}-r_{2}uv,$$

where *n*_1_ > 2, and *m*_1_, *n*_3_ and *m*_3_ are non-negative but are as yet otherwise unconstrained. Supposing the existence of a uniform steady state, we find

$$\det(J^{*})=2r_{1}r_{2}(m_{1}-(n_{1}-2)){\left(u^{*}\right)}^{2},$$

and so for linear stability we require *m*_1_ > *n*_1_ − 2 > 0 and thus the qualitative type of the 2*U* reaction is determined. Solving the steady-state equations, we find a positive steady state only if *n*_3_ > *m*_3_,

$$\begin{array}{rl} u^{*} & =\sqrt{\frac{r_{3}}{r_{1}}}\sqrt{\frac{n_{3}-m_{3}}{m_{1}-(n_{1}-2)}},\;\;\\ v^{*} & =\frac{\sqrt{r_{3}r_{1}}}{r_{2}}\;\frac{n_{3}m_{1}-m_{3}(n_{1}-2)}{\sqrt{\left(n_{3}-m_{3})(m_{1}-(n_{1}-2)\right)}}, \end{array}$$

and this is then the unique positive steady state. This yields

$$J_{12}^{*}=J_{22}^{*}=-r_{2}\sqrt{\frac{r_{3}}{r_{1}}}\sqrt{\frac{n_{3}-m_{3}}{m_{1}-(n_{1}-2)}}$$

and

$$J_{21}^{*}=2r_{1}m_{1}u^{*}-r_{2}v^{*}>2r_{1}(n_{1}-2)u^{*}-r_{2}v^{*}=J_{11}^{*},$$

hence J* fully matches the required sign pattern if and only if $J_{11}^{*}>0$,

$$\begin{array}{rl} J_{11}^{*}>0\;\Leftrightarrow & \;2(n_{1}-2)u^{*}>r_{2}v^{*}\\ \Leftrightarrow & \;\;(n_{3}-m_{3})(n_{1}-2)>n_{3}(m_{1}-(n_{1}-2)). \end{array}$$

The last condition for Turing instability is the trace condition (2.3) for linear stability of the homogeneous state to uniform perturbations,

$$\mathrm{tr}(J^{*})\;<0\;\;\;\Leftrightarrow \;\;\;\frac{r_{2}}{r_{1}}\;>\frac{\left(n_{3}-m_{3})(n_{1}-2)-n_{3}(m_{1}-(n_{1}-2)\right)}{n_{3}-m_{3}},$$

which may be satisfied for any choices of stoichiometric product coefficients if *r*_2_ is sufficiently large. Thus, this choice of third reactant combination yields two qualitatively distinct Turing-unstable reaction schemes,

$$\{\begin{array}{c} 2U\overset{r_{1}}{⟶}n_{1}^{\prime \prime }U+m_{1}V\\ \;U+V\overset{r_{2}}{⟶}\emptyset \\ \;\emptyset \overset{r_{3}}{⟶}n_{3}U \end{array}\mathrm{and}\;\;\;\;\{\begin{array}{c} 2U\overset{r_{1}}{⟶}n_{1}^{\prime \prime }\;U+m_{1}V\\ \;U+V\overset{r_{2}}{⟶}\emptyset \\ \;\emptyset \overset{r_{3}}{⟶}n_{3}U+m_{3}V \end{array},$$

where $n_{1}^{\prime \prime }>2$, and *m*_1_, *n*_3_, *m*_3_ > 0. The first is Turing-unstable if and only if

$$2(n_{1}^{\prime \prime }-2)>m_{1}>n_{1}^{\prime \prime }-2,$$

while the second is Turing-unstable if and only if

$$\begin{array}{r} m_{1}>n_{1}^{\prime \prime }-2,\;\;n_{3}>m_{3}\;\;\;\mathrm{and}\\ 2(n_{1}^{\prime \prime }-2)n_{3}>m_{1}n_{3}+(n_{1}^{\prime \prime }-2)m_{3}. \end{array}$$

### 4.2. Third reaction with reactant *U*

If we choose the third reaction to have reactant *U*, existence of a positive steady state again requires that the bimolecular *U* + *V* reaction must decrease the number of particles of species *V*, determining the qualitative type of this reaction. We may then write our reaction scheme as

$$\{\begin{array}{c} 2U\overset{r_{1}}{⟶}n_{1}U+m_{1}V\\ \;U+V\overset{r_{2}}{⟶}\emptyset \\ U\overset{r_{3}}{⟶}n_{3}U+m_{3}V \end{array},$$

with corresponding reaction terms

$$F(u,v)=r_{3}(n_{3}-1)u+r_{1}(n-2)u^{2}-r_{2}uv\;$$

and

$$G(u,\;v)=r_{3}m_{3}u+r_{1}m_{1}u^{2}-r_{2}uv,$$

where *n*_1_ > 2, and *m*_1_, *n*_3_ and *m*_3_ are non-negative but are (for the moment) otherwise unconstrained. Supposing the existence of a uniform steady state, we find

$$\det(J^{*})=r_{1}r_{2}(m_{1}-(n_{1}-2)){\left(u^{*}\right)}^{2},$$

and so for linear stability we require *m*_1_ > *n*_1_ − 2 > 0, determining the qualitative type of the 2*U* reaction. Solving the steady-state equations, we find a positive steady state only if *n*_3_ − 1 > *m*_3_,

$$\begin{array}{rl} u^{*} & =\left(\frac{r_{3}}{r_{1}}\right)\frac{(n_{3}-1)-m_{3}}{m_{1}-(n_{1}-2)},\;\;\\ v^{*} & =\left(\frac{r_{3}}{r_{2}}\right)\;\frac{m_{1}(n_{3}-1)-(n_{1}-2)m_{3}}{m_{1}-(n_{1}-2)}, \end{array}$$

and this is the only non-negative steady state. The signs of J* are guaranteed to match those required for type-I patterns, and so the last condition to satisfy is the trace condition (2.3),

$$\mathrm{tr}(J^{*})\;<0\;\;\;\Leftrightarrow \;\;\;\frac{r_{2}}{r_{1}}\;>n_{1}-2,$$

which may be satisfied for any choice of *n*_1_ if *r*_2_ is sufficiently large. Thus we have two qualitatively distinct Turing-unstable reaction schemes,

$$\{\begin{array}{c} \;2U\overset{r_{1}}{⟶}n_{1}^{\prime \prime }U+m_{1}V\\ \;U+V\overset{r_{2}}{⟶}\emptyset \\ \;U\overset{r_{3}}{⟶}n_{3}^{\prime }U \end{array}\;\mathrm{and}\;\{\begin{array}{c} \;2U\overset{r_{1}}{⟶}n_{1}^{\prime \prime }U+m_{1}V\\ \;U+V\overset{r_{2}}{⟶}\emptyset \\ \;U\overset{r_{3}}{⟶}n_{3}^{\prime }U+m_{3}V \end{array},$$

where $n_{1}^{\prime \prime }>2$, $n_{3}^{\prime }>1$, and *m*_1_, *m*_3_ > 0. The first is Turing-unstable if and only if $m_{1}>n_{1}^{\prime \prime }-2$, while the second is Turing-unstable if and only if $m_{1}>n_{1}^{\prime \prime }-2$ and $n_{3}^{\prime }-1>m_{3}$.

### 4.3. Third reaction with reactant *V*

Choosing the third reaction to be a first-order interaction with reactant *V* yields the largest number of options for Turing-unstable reaction schemes. For convenience, we divide this case into three sub-cases according to the qualitative type of the *U* + *V* reaction.

#### 4.3.1. Interspecific reaction removing both species (U+V→∅)

In the first sub-case, we may write the reaction scheme as

$$\{\begin{array}{c} 2U\overset{r_{1}}{⟶}n_{1}U+m_{1}V\\ \;U+V\overset{r_{2}}{⟶}\emptyset \\ V\overset{r_{3}}{⟶}n_{3}U+m_{3}V \end{array},$$

with corresponding interaction terms

$$F(u,\;v)=r_{3}n_{3}v+r_{1}(n_{1}-2)u^{2}-r_{2}uv\;$$

and

$$G(u,\;v)=r_{3}(m_{3}-1)v+r_{1}m_{1}u^{2}-r_{2}uv,$$

where *n*_1_ > 2, and *m*_1_, *n*_3_ and *m*_3_ are non-negative but are otherwise as yet unconstrained. These interaction terms admit a unique non-zero steady state,

$$\begin{array}{rl} u^{*} & =\frac{r_{3}}{r_{2}}\frac{m_{1}n_{3}-(n_{1}-2)(m_{3}-1)}{m_{1}-(n_{1}-2)},\\ v^{*} & =\frac{r_{1}r_{3}}{r_{2}^{2}}\;\frac{{\left(m_{1}n_{3}-(n_{1}-2)(m_{3}-1)\right)}^{2}}{\left(m_{1}-(n_{1}-2))(n_{3}-(m_{3}-1)\right)}, \end{array}$$

which yields

$$\det(J^{*})=r_{1}r_{2}(m_{1}-(n_{1}-2)){\left(u^{*}\right)}^{2}.$$

For linear stability, we require *m*_1_ > *n*_1_ − 2, and then for positivity of the steady state we require *n*_3_ > *m*_3_ − 1. The signs of $J_{12}^{*}$ and $J_{22}^{*}$ match those required for type-I patterns; the steady state admits $J_{21}^{*}>J_{11}^{*}$, and so J* fully matches the required sign pattern if and only if $J_{11}^{*}>0$,

$$J_{11}^{*}>0\;\Leftrightarrow \;\;2(n_{1}-2)n_{3}>m_{1}n_{3}+(n_{1}-2)(m_{3}-1).$$

In particular, if *n*_3_ = 0 then we require *m*_3_ = 0. The trace condition (2.3) is

$$\frac{r_{2}}{r_{1}}>\frac{\left(m_{1}n_{3}-(n_{1}-2)(m_{3}-1))(2(n_{1}-2)n_{3}-m_{1}n_{3}-(n_{1}-2)(m_{3}-1)\right)}{m_{1}(m_{1}-(n_{1}-2)){\left(n_{3}-(m_{3}-1)\right)}^{2}},$$

which can be satisfied for any choice of the stoichiometric product coefficients by taking *r*_2_ to be sufficiently large. Thus, this case yields four qualitatively distinct Turing-unstable reaction schemes,

$$\{\begin{array}{c} 2U\overset{r_{1}}{⟶}n_{1}^{\prime \prime }\;U+m_{1}V\\ \;U+V\overset{r_{2}}{⟶}\emptyset \end{array},$$

together with one of

$$\begin{array}{r} (i)\;\;\{V\overset{r_{3}}{\to }\emptyset ,\;\;(\mathrm{ii})\;\;\{V\overset{r_{3}}{⟶}n_{3}U,\\ (\mathrm{iii})\;\;\{V\overset{r_{3}}{⟶}n_{3}U+V,\;\;(\mathrm{iv})\;\;\{V\overset{r_{3}}{⟶}n_{3}U+m_{3}^{\prime }V, \end{array}$$

where $n_{1}^{\prime \prime }>2$, $m_{3}^{\prime }>1$ and *m*_1_, *n*_3_ > 0. From the sign constraint on $J_{11}^{*}$, option (i) is Turing-unstable if and only if

$$m_{1}>n_{1}^{\prime \prime }-2;$$

option (ii) is Turing-unstable if and only if

$$m_{1}>n_{1}^{\prime \prime }-2\;\;\;\;\mathrm{and}\;\;\;\;(n_{1}^{\prime \prime }-2)(2n_{3}+1)>m_{1}n_{3};$$

option (iii) is Turing-unstable if and only if

$$m_{1}>n_{1}^{\prime \prime }-2\;\;\;\;\mathrm{and}\;\;\;\;2(n_{1}^{\prime \prime }-2)>m_{1};$$

option (iv) is Turing-unstable if and only if

$$\begin{array}{r} m_{1}>n_{1}^{\prime \prime }-2,\;\;\;n_{3}>m_{3}^{\prime }-1\;\;\;\;\mathrm{and}\\ 2(n_{1}^{\prime \prime }-2)n_{3}>m_{1}n_{3}+(n_{1}^{\prime \prime }-2)({m^{\prime }}_{3}-1). \end{array}$$

#### 4.3.2. Interspecific reaction removing species *U* and preserving species *V* (*U* + *V* → *V*)

In the second sub-case, existence of a positive steady state requires that the interaction with reactant *V* must reduce the number of particles of species *V* while the 2*U* reaction must increase the number of particles of *V*. We may then write our reaction scheme as

$$\{\begin{array}{c} 2U\overset{r_{1}}{⟶}n_{1}U+m_{1}V\\ \;U+V\overset{r_{2}}{⟶}V\\ \;V\overset{r_{3}}{⟶}n_{3}U \end{array},$$

with corresponding interaction terms

$$F(u,\;v)=r_{3}n_{3}v+r_{1}(n_{1}-2)u^{2}-r_{2}uv\;$$

and

$$G(u,\;v)=-r_{3}v+r_{1}m_{1}u^{2},$$

where *n*_1_ > 2 and *m*_1_ > 0 and *n*_3_ is non-negative but is otherwise as yet unconstrained. This yields a unique positive steady state,

$$u^{*}=\frac{r_{3}}{r_{2}}\frac{m_{1}n_{3}+n_{1}-2}{m_{1}},\;\;v^{*}=\frac{r_{1}r_{3}}{r_{2}^{2}}\;\frac{{\left(m_{1}n_{3}+n_{1}-2\right)}^{2}}{m_{1}},$$

which satisfies det(J*) > 0, $J_{12}^{*}<0$, $J_{21}^{*}>0$ and $J_{22}^{*}<0$. We find

$$J_{11}^{*}=\frac{r_{1}r_{3}}{r_{2}}(n_{1}-2-m_{1}n_{3})\frac{m_{1}n_{3}+n_{1}-2}{m_{1}},$$

and thus the sign pattern condition on J* is satisfied if and only if *n*_1_ − 2 > *m*_1_*n*_3_. Lastly, the trace condition (2.3) yields

$$\mathrm{tr}(J^{*})\;<0\;\Leftrightarrow \;\;\frac{r_{2}}{r_{1}}>\frac{{\left(n_{1}-2\right)}^{2}-m_{1}^{2}n_{3}^{2}}{m_{1}},$$

which may be satisfied for any choice of the stoichiometric product coefficients provided that *r*_2_ is sufficiently large. Thus, this case yields two qualitatively distinct Turing-unstable reaction schemes,

$$\{\begin{array}{c} 2U\overset{r_{1}}{⟶}n_{1}^{\prime \prime }\;U+m_{1}V\\ \;U+V\overset{r_{2}}{⟶}V\;\\ \;V\overset{r_{3}}{⟶}\emptyset \; \end{array}\;\mathrm{and}\;\{\begin{array}{c} 2U\overset{r_{1}}{⟶}n_{1}^{\prime \prime }\;U+m_{1}V\\ \;U+V\overset{r_{2}}{⟶}V\;\\ \;V\overset{r_{3}}{⟶}n_{3}U\; \end{array},$$

where $n_{1}^{\prime \prime }>2$, and *m*_1_, *n*_3_ > 0. The first is Turing-unstable for all choices of $n_{1}^{\prime \prime }$ and *m*_1_, while the second is Turing-unstable if and only if $n_{1}^{\prime \prime }-2>m_{1}n_{3}$.

#### 4.3.3. Interspecific reaction removing species *U* and increasing species *V* ($U+V\to m_{2}^{\prime }V$)

In the third sub-case, existence of a positive steady state requires that the interaction with reactant *V* must reduce the number of particles of species *V*. We may then write our reaction scheme as

$$\{\begin{array}{c} 2U\overset{r_{1}}{⟶}n_{1}U+m_{1}V\\ \;U+V\overset{r_{2}}{⟶}m_{2}V\\ \;V\overset{r_{3}}{⟶}n_{3}U \end{array},$$

with corresponding interaction terms

$$F(u,\;v)=r_{3}n_{3}v+r_{1}(n_{1}-2)u^{2}-r_{2}uv\;$$

and

$$G(u,\;v)=-r_{3}v+r_{1}m_{1}u^{2}+r_{2}(m_{2}-1)uv,$$

where *n*_1_ > 2, *m*_2_ > 1 and *m*_1_, *n*_3_ are non-negative but are as yet otherwise unconstrained. Supposing the existence of a positive steady state, to satisfy $J_{12}^{*}<0$ we must have

$$r_{3}n_{3}<r_{2}u^{*},$$

and to satisfy $J_{22}^{*}<0$ we must have

$$r_{2}(m_{2}-1)u^{*}<r_{3}.$$

These two inequalities can only be satisfied simultaneously if *n*_3_ = 0, thus the third reaction is of the type V→∅. Solving the steady-state equations we find a unique positive steady state,

$$\begin{array}{rl} u^{*} & =\frac{r_{3}}{r_{2}}\frac{n_{1}-2}{m_{1}+(n_{1}-2)(m_{2}-1)},\\ v^{*} & =\frac{r_{1}r_{3}}{r_{2}^{2}}\;\frac{{\left(n_{1}-2\right)}^{2}}{m_{1}+(n_{1}-2)(m_{2}-1)}. \end{array}$$

At this steady state, we have det(J*) > 0 and

$$\mathrm{Tr}(J^{*})\;<0\;\Leftrightarrow \;\frac{r_{2}}{r_{1}}>\frac{{\left(n_{1}-2\right)}^{2}}{m_{1}}.$$

The signs of $J_{11}^{*}$, $J_{12}^{*}$ and $J_{21}^{*}$ match those required for type-I patterns, while $J_{22}^{*}<0$ if and only if *m*_1_ > 0—which finally determines the qualitative type of the reaction with reactant combination 2*U*. Accordingly, this yields only one qualitative type of Turing-unstable reaction scheme,

$$\{\begin{array}{c} 2U\overset{r_{1}}{⟶}n_{1}^{\prime \prime }\;U+m_{1}V\\ \;U+V\overset{r_{2}}{⟶}m_{2}^{\prime }V\\ \;V\overset{r_{3}}{⟶}\emptyset \end{array},$$

which is Turing-unstable for all choices of $n_{1}^{\prime \prime }>2$, $m_{2}^{\prime }>1$ and *m*_1_ > 0.

### 4.4. Third reaction with reactant combination 2*V*

We now turn to the case in which the third reaction has the reactant combination 2*V* which yields the interaction terms

$$F(u,\;v)=a_{4}u^{2}+a_{5}uv+a_{6}v^{2}\;$$

and

$$G(u,\;v)=b_{4}u^{2}+b_{5}uv+b_{6}v^{2},$$

and supposing the existence of a positive steady state (u*, v*) we find

$$J^{*}=\left(\;\begin{array}{cc} 2a_{4}u^{*}+a_{5}v^{*} & a_{5}u^{*}+2a_{6}v^{*}\\ 2b_{4}u^{*}+b_{5}v^{*} & b_{5}u^{*}+2b_{6}v^{*} \end{array}\;\right)=\left(\;\begin{array}{cc} -\frac{v^{*}}{u^{*}}(a_{5}u^{*}+2a_{6}v^{*}) & a_{5}u^{*}+2a_{6}v^{*}\\ -\frac{v^{*}}{u^{*}}(b_{5}u^{*}+2b_{6}v^{*}) & b_{5}u^{*}+2b_{6}v^{*} \end{array}\;\right),$$

and thus det(J*) = 0. Hence we reject this choice of third reactant combination as it violates the condition of strict linear stability of (u*, v*), and thus offers no Turing-unstable reaction schemes.

### 4.5. Linear stability boundaries for type-I minimal schemes

**Figure 5.**
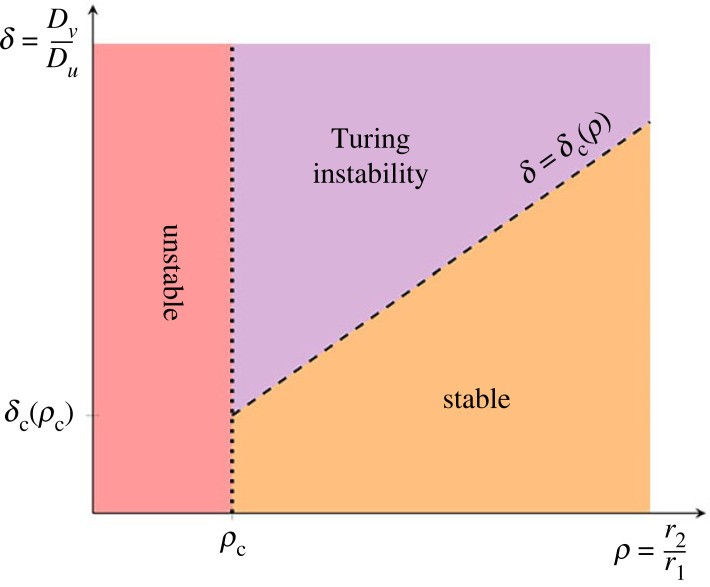
Phase diagram of the linear stability of the homogeneous steady state for the minimal Turing-unstable reaction schemes for patterns of type I. The parameters *r*_1_ and *r*_2_ are the rate constants for the 2*U* → · · · and *U* + *V* → · · · reactions, respectively. The values of *ρ_c_* and *δ_c_*(*ρ*)/*ρ* depend on the choice of minimal reaction scheme and the stoichiometric product coefficients, as collated in section G of the electronic supplementary material. When *ρ* < *ρ_c_* (region marked ‘unstable', pink) the spatially uniform equilibrium is unstable to uniform disturbances. In the Turing instability regime a spatial pattern emerges as *δ* increases above *δ_c_*.

A complete analysis of the linear instability behaviour for the minimal type-I schemes is provided in electronic supplementary material, text section B. Remarkably, it turns out that the minimal schemes of type I can all be described in terms of a single regime diagram, shown in figure 5. The first thing to note is that each type-I minimal scheme admits a unique positive homogeneous steady state; bistability is not possible. Next, our results reveal that the onset of the Turing instability depends on only two parameter ratios: the ratio of diffusivities *δ*: = *D_v_*/*D_u_* (a crucial parameter in determining the existence of instability in Turing's classic analysis) and the ratio *ρ* := *r*_2_/*r*_1_ where *r*_1_ and *r*_2_ are, respectively, the rates of the intraspecific (2*U* → · · ·) and interspecific (*U* + *V* → · · ·) bimolecular reactions, having first non-dimensionalized these rates by fixing the rate of the third reaction to be unity. In every case Turing instability arises only when the ratio *ρ* := *r*_2_/*r*_1_ lies above a threshold value *ρ_c_*, and then when *δ* > *δ_c_*(*ρ*) where this boundary depends linearly on *ρ* and has a gradient that can be computed explicitly in terms of the stoichiometric coefficients in a particular reaction scheme. This reduction to a single regime diagram allows direct comparisons between the 11 cases; for example, computation of the lowest possible value for the minimum diffusivity ratio *δ_c_* as the stoichiometric coefficients change.

A canonical criticism of the Turing mechanism is that the PDE system (2.2) may require an order of magnitude (or greater) disparity between the diffusivities *D_u_* and *D_v_* in order to satisfy the conditions for Turing instability. This is rarely observed in real-world systems where interacting species typically diffuse through the same medium and are often of comparable sizes—thereby if motion is accurately modelled by isotropic diffusion, we expect species to have similar diffusivities. As a reference example, in modelling the chlorite–iodide–malonic acid–starch system, Lengyel & Epstein [15] determined the diffusivities of the interacting species to differ by no more than a factor of 2: in practice, a binding gel was introduced to drastically reduce the diffusivity of one species, and more recent work [27] has analysed the dynamical effects of allowing reactants to bind to an immobile substrate. With this in mind, we might ask what the smallest possible value of *δ_c_*(*ρ_c_*) is for each class: these being the smallest necessary disparities in the diffusivity parameters required for Turing instability. In electronic supplementary material, text section C, we tabulate the functions *ρ_c_*(***n***, ***m***) and $\sqrt{H(n,m)}$ for each class, given by

$$\begin{array}{rl} \rho_{c}(n,m) & =\frac{J_{11}^{*}}{-J_{22}^{*}}\rho \;\;\;\;\mathrm{and}\\ \sqrt{H(n,m)} & =\frac{1}{J_{11}^{*}}\;\left(\sqrt{\det(J^{*})}+\;\sqrt{-J_{12}^{*}J_{21}^{*}}\right)\rho^{-\frac{1}{2}}, \end{array}$$

respectively. We also present there the corresponding functions $\sqrt{\delta_{c}(\rho_{c})}=\sqrt{H\rho_{c}}$. In general, $\sqrt{\delta_{c}(\rho_{c})}$ does not have a defined minimum value over the integers {*n_i_*, *m_i_*} within the constraints for Turing instability, but is monotonically decreasing as one or more of the variables becomes arbitrarily large. For each class *δ_c_*(*ρ_c_*) does have an infimum, and these are also presented. For all but three classes, it transpires that inf(*δ_c_*(*ρ_c_*)) = 1, but we reiterate that this is in general an infimum and not an achievable minimum; for *δ_c_* to take values arbitrarily close to its infimum, it may be necessary for one or more of the stoichiometric product coefficients to be arbitrarily large, which may present a challenge in terms of a physical or biological interpretation of such a reaction scheme.

**Figure 6.**
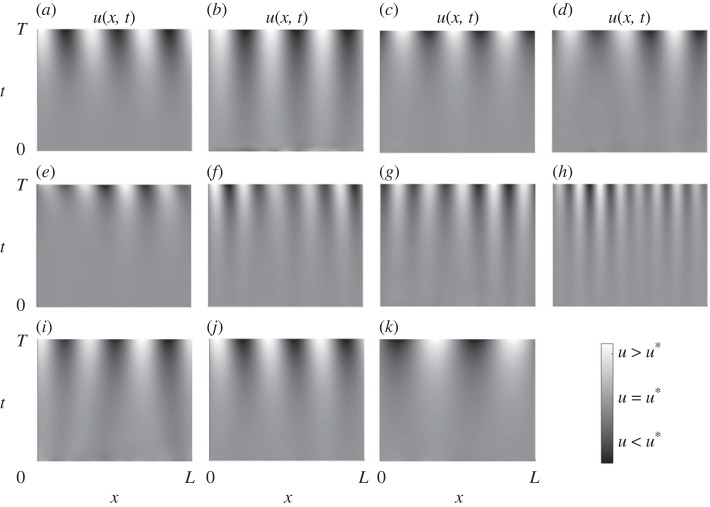
One-dimensional numerical simulations of example minimal Turing-unstable reaction schemes from each class for patterns of type I. Heatmap plots of the concentration *u*(*x*, *t*): spatial coordinate is plotted on the horizontal axis with time increasing up the vertical axis. Each simulation uses the same spatial scale, but time scales vary; full simulation details and parameter values are provided in electronic supplementary material, text section E.

**Figure 7.**
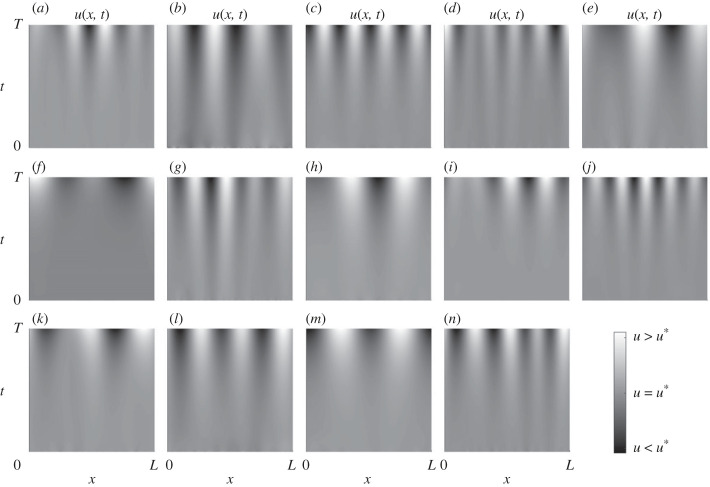
One-dimensional numerical simulations of example minimal Turing-unstable reaction schemes from each class for patterns of type II. Heatmap plots of the concentration *u*(*x*, *t*): spatial coordinate is plotted on the horizontal axis with time increasing up the vertical axis. Each simulation uses the same spatial scale, but time scales vary; full simulation details and parameter values are provided in electronic supplementary material, text section E.

Numerical simulations of the onset of instability in example Turing-unstable schemes from each class for type-I are shown in figure 6, and similarly for type II in figure 7. All simulation parameters and details are given in electronic supplementary material, text section E.

## 5. Outlook

In summary, we have, for the first time, enumerated all possible Turing-unstable reaction schemes with the fewest number of elementary (i.e. at most biomolecular) reactions under our parsimonious and physically reasonable modelling assumptions. Given that there are over 5000 possible qualitatively distinct schemes of three elementary reactions, and over 45 000 schemes for four reactions, the numbers of minimal Turing-unstable schemes are surprisingly small. This demonstrates that physical constraints (such as the existence of a spatially uniform equilibrium at positive concentrations) combined with the Turing instability conditions can indeed be satisfied but require specific, carefully designed reaction schemes.

In contrast to existing popular baseline models of pattern formation which typically employ third-order interactions (see the Brusselator [17], Schnakenberg [18] or Gray–Scott [19] models) or rational nonlinearities (for example, the Gierer–Meinhardt [28] short-range activator long-range inhibitor models), we have established the complete catalogue of minimal models with at most quadratic polynomial nonlinearities that are sufficient to exhibit Turing's reaction–diffusion instability.

Where previous studies of minimal Turing-unstable models have employed more context-specific assumptions and required computationally automated parameter sweeping to approximately identify Turing instabilities [29], our assumptions yield models with direct particle-scale interpretations which are also amenable to exact analysis, enabling our definitive list of minimal reaction schemes, which we propose as the simplest theoretical models for Turing instability. Our analysis allows us to separate out the distinct contributions made by (i) different kinds of particle interactions (i.e. reagents), (ii) reaction rates and (iii) stoichiometry of the reaction products, in determining where in parameter space we expect patterns to arise. This clear separation of these three elements of the reaction scheme is novel in its clarity.

Although it has long been assumed that an auto-catalytic reaction is necessary for Turing instability, our analysis is novel in being able to confirm this and, further, to identify the following essential particle interactions: (i) a multi-molecular reaction for species *U* that increases the number of particles of *U* and (ii) a multi-molecular interspecific reaction which decreases the number of particles of one or both species. For type-I patterns, there must also be at least one reaction of zeroth or first order, while for type-II patterns, there must be both a first-order reaction that decreases the number of particles of *U*, and also another reaction that spontaneously generates particles, or has only particles of species *V* as its reactants. These stand as generic design principles with which we might better identify natural Turing-unstable systems, or design artificial ones [30], while addressing systematically issues of robustness and genericity of mechanism.

All the analysis in this paper is concerned with linear theory—applicable to these reaction–diffusion systems only when perturbations to the homogeneous steady state are small, and typically only for short times. Nonlinear theory is essential in order to be able to understand, and to predict, the dynamics at later times [5,31]. In particular, nonlinear theory is needed to understand whether or not, for particular parameter values close to the Turing bifurcation point, stable small-amplitude periodic patterns exist. The details of the nonlinear behaviour will also depend significantly on the choices for parameter values: the reaction stoichiometry, reaction rates and diffusion rates.

**Figure 8.**
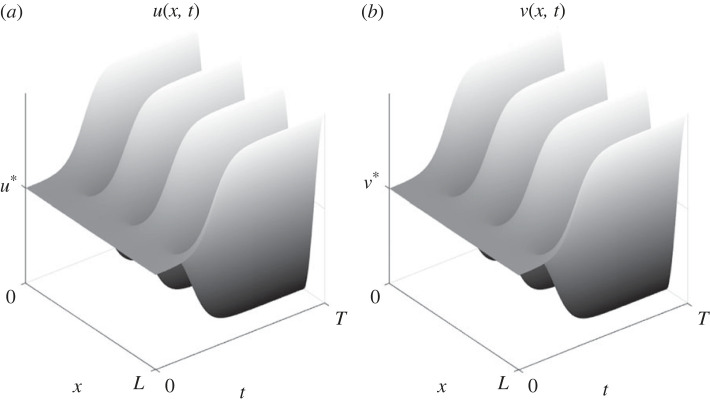
One example of a stable saturating patterned state from a type-I instability. Heatmap plots of the concentrations (*a*) *u*(*x*, *t*) and (*b*) *v*(*x*, *t*) relative to the homogeneous steady-state values u*, v*: the reaction scheme used here is from class a. of the type-I minimal schemes with stoichiometric product coefficients $\left(n_{1}^{\prime \prime },n_{3},m_{3})=(4,1,3\right)$. For this simulation *ρ* = 1.2 and *δ* = 15; full simulation details and parameter values are provided in electronic supplementary material, text section E.

An example of a set of parameter values for which one minimal reaction scheme exhibits a small-amplitude stable periodic pattern is shown in figure 8. A typical weakly nonlinear analysis in one spatial dimension close to the Turing bifurcation point, introducing long space and time scales (*X*, *T*), generically allows for the derivation of a real Ginzburg–Landau equation

$$\partial_{T}A=\;\partial_{XX}A+\mu A-\kappa A{\left|A\right|}^{2}$$

for the leading-order amplitude *A*(*X*, *T*) of a monochromatic perturbation from the homogeneous steady state, where the coefficient *κ* is a function of the reaction rates and stoichiometry. If *κ* > 0, then, generically, we would expect stable small amplitude patterned states to exist close to the Turing bifurcation point. Preliminary analysis of the type-I minimal schemes reveals that the sign of *κ* depends in subtle ways on the choice of stoichiometry. Each of the classes of type-I minimal scheme contain some Turing-unstable reaction schemes for which *κ* > 0, except for classes c. and d. for which *κ* appears to uniformly vanish—and so perhaps a different scaling is required in the nonlinear analysis. For the classes of type-II minimal schemes, and in two or more spatial dimensions, the analysis is more complicated. Given the high-dimensional nature of the parameter space, and that the nonlinear behaviours will differ from one reaction scheme to the next, we will defer presentation of further results to future work. A much more thorough exploration of the nonlinear behaviour of both the type-I and type-II minimal schemes is, however, clearly warranted, but lies beyond the scope of this paper due to our focus here on the purely linear instability problem. We will return to the question of nonlinear behaviour in future work and hope there to carry out the much more comprehensive investigation that is required.

The literature on the general analysis of stoichiometry-based criteria for particular dynamic behaviours in reaction networks is large [32–34]. Providing detailed connections between our results and those more general criteria is beyond the scope of this work. Here we have constructed the complete set of the simplest possible reaction schemes rather than an analysis of the most general situation. Our minimal schemes can naturally be embedded within more complicated sets of reactions involving more species, or intermediate products; however, the inclusion of even one more species makes the range of patterning behaviour much richer [35,36]. Naturally, the minimal schemes presented here do not necessarily capture the full dynamics of any real system. The converse problem of reducing a complex model to a smaller one that still exhibits Turing instability entails its own difficulties [37]. Our analysis provides significant new clarity for a long-standing and widely used mechanism; it opens up promising new lines of theoretical work since it enables a principled and complete study of the stochastic dynamics of these minimal particle-based schemes as the simplest settings in which one can compare the results of stochastic analysis and simulation with results for the mass-action models. This will allow us to understand, with much greater clarity, the effects of stochasticity on Turing pattern formation in these minimal, and physically achievable, cases.

More broadly, as both deterministic and stochastic models for Turing patterns continue to fascinate and provide food for thought in biology, physics, engineering and many other fields besides, we expect that study of these new minimal models will aid the development of concepts and quantitative modelling across many areas of natural science.

## Data Availability

Details of the code used to produce figures 6–8 are provided in the electronic supplementary material. Code files are available from the Zenodo digital repository: doi:10.5281/zenodo.10590813 [38]. Supplementary material is available online [39].
